# Online Fabric Defect Inspection Using Smart Visual Sensors

**DOI:** 10.3390/s130404659

**Published:** 2013-04-09

**Authors:** Yundong Li, Jingxuan Ai, Changqing Sun

**Affiliations:** 1 School of Information Engineering, North China University of Technology, Beijing 100041, China; E-Mail: sunchangqing134@hotmail.com; 2 Development Competence Center, France Telecom R&D Beijing Center, Beijing 100190, China; E-Mail: Jingxuan.ai@orange.com

**Keywords:** machine vision, fabric defect inspection, smart visual sensor, wavelet transform, mathematical morphology filter

## Abstract

Fabric defect inspection is necessary and essential for quality control in the textile industry. Traditionally, fabric inspection to assure textile quality is done by humans, however, in the past years, researchers have paid attention to PC-based automatic inspection systems to improve the detection efficiency. This paper proposes a novel automatic inspection scheme for the warp knitting machine using smart visual sensors. The proposed system consists of multiple smart visual sensors and a controller. Each sensor can scan 800 mm width of web, and can work independently. The following are considered in dealing with broken-end defects caused by a single yarn: first, a smart visual sensor is composed of a powerful DSP processor and a 2-megapixel high definition image sensor. Second, a wavelet transform is used to decompose fabric images, and an improved direct thresholding method based on high frequency coefficients is proposed. Third, a proper template is chosen in a mathematical morphology filter to remove noise. Fourth, a defect detection algorithm is optimized to meet real-time demands. The proposed scheme has been running for six months on a warp knitting machine in a textile factory. The actual operation shows that the system is effective, and its detection rate reaches 98%.

## 1. Introduction

Defect inspection is a quality control process that identifies and locates deficiencies in the fabric manufactured in the textile industry. Traditionally, defects are detected by human eyes, but the efficiency of the manual method is low because of eye fatigue. Hence, an automatic inspection system becomes an effective way to improve textile quality because of the progress of machine vision technology. Research in this field has been carried out, and some PC-based prototype systems have been developed.

Abouelela [1] proposed a visual detection system that consisted of a camera, frame grabber and a computer. Defects were identified and located through image binarization with a fixed threshold. Saeidi [2] developed a visual inspection system for a circular knitting machine, which comprised a CMOS camera with 640 × 320 resolution and a computer, while the Garbor wavelet was used in the detection algorithm.

Rocco [3] proposed a real-time visual detection method based on a neural network. This method can accomplish real-time detection and classification of the most frequently occurring types of defects in knitted fabrics, and its detection rate was 93%. Mak [4] built a prototype system in the lab, and the system consisted of lighting, line scan cameras, a frame grabber, and a computer. The Gabor wavelet was used in the detection algorithm. Sun [5] proposed an adaptive inspection system based on a PCNN neural network, which had area scan cameras with resolution of 800 × 600 and a computer. Experiments showed the effectiveness of his method for plain and interlocked weft-knitted fabrics with holes, dropped stitches, and course mark defects.

**Figure 1.**
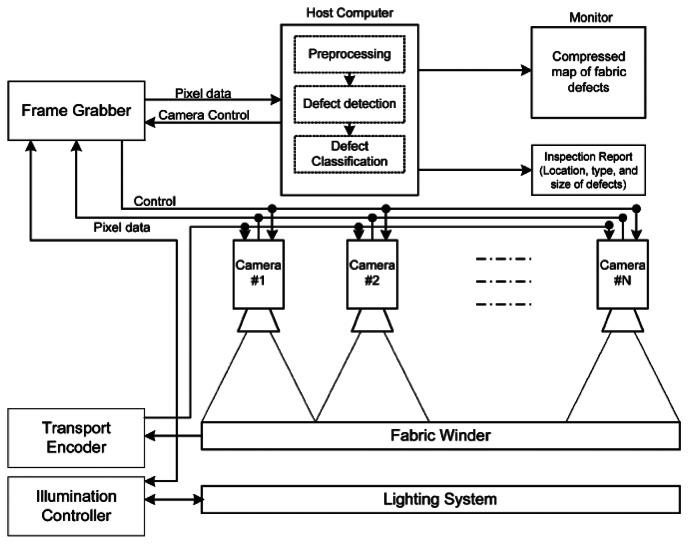
PC-based fabric inspection system.

All these schemes employed PC-based architectures that consisted of a lighting system, cameras, frame grabbers, and host computers. Figure 1 illustrates this scheme [6]. The computer is the central unit in this architecture. Fabric images are captured through a graphic card, and are fed to the CPU to run the detection algorithms, and the results are output through the control unit. Although the PC-based inspection systems have powerful computational capabilities, their disadvantages are obvious, such as high cost, big size, high power dissipation, and so on.

Along with the upgrading of the computational capability of embedded DSPs, integrating the image sensor together with the DSP is possible in the form of a smart visual sensor. This study proposes an automatic inspection scheme using smart visual sensors, which are used in the detection of fabric defects in a warp knitting machine.

## 2. System Architecture

**Figure 2.**
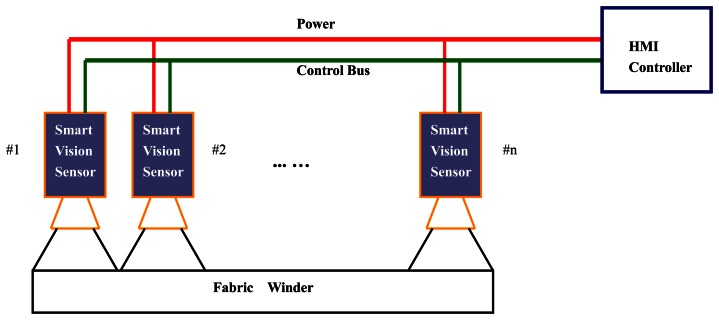
Automatic inspection system using smart visual sensors.

The proposed inspection scheme, which is based on smart visual sensors, is illustrated in Figure 2.

### 2.1. Smart Visual Sensor

Traditional industrial cameras collect images without any analysis on those images. When a camera is integrated with a high performance embedded processor where detection algorithms are running, it becomes a smart visual sensor. The advantages of the smart visual sensor are obvious, and include small size, ease of installation, low power consumption, cheap cost, *etc*. Moreover, each smart visual sensor works independently, which means the breakdown of single sensor will not affect the others. In practice, multiple smart visual sensors are arranged in parallel across the web to be scanned. Each device covers about 800 mm width of web and six sensors are needed for a typical 210-inch-wide warp knitting machine. All of the normally closed relay nodes of sensors are connected in series to the Human Machine Interface (HMI) controller. The HMI controller will then be informed when any sensor has detected a defect. The parts of the smart visual sensor are described in the following sections, from the hardware scheme and software architecture to the detection algorithm.

### 2.2. Hardware Scheme

**Figure 3.**
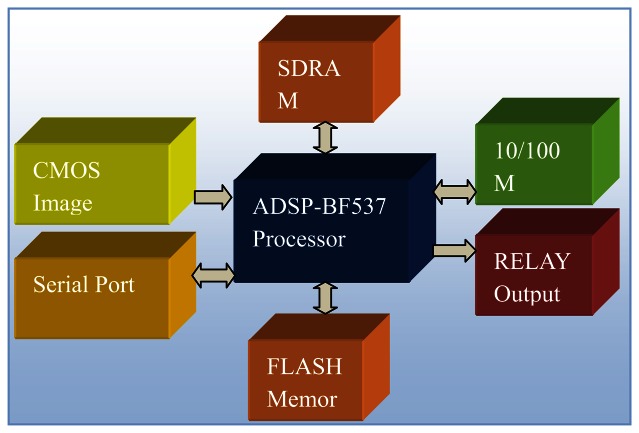
Hardware diagram of smart visual sensor.

The smart visual sensor consists of the CMOS image sensor, embedded DSP, SDRAM memory, FLASH memory, Ethernet interface, RS232/485 serial port, and relay control circuits. The block diagram is shown in Figure 3.

- **Processor:** A BF537 DSP with 600 MHz clock speed is chosen as the host processor. This processor is the member of ADI Blackfin family products, which incorporates the Micro Signal Architecture (MSA). Blackfin processors combine a dual-MAC, state-of-the-art signal processing engine, the advantages of a clean and orthogonal RISC-like microprocessor instruction set, single-instruction, and multiple-data (SIMD) multimedia capabilities into a single instruction-set architecture. Hence, the processor is suitable for applications such as smart sensors that need both low power consumption and high computing capability.
- **Image sensor:** A 2-megapixel CMOS image sensor with 1,600 × 1,200 resolution is employed. To improve the processing speed, a sub-window of 1,600 × 100 is cropped from the center of the field of view (FOV). The CMOS sensor outputs the data in YUV422 format, which is transferred into the memory of the DSP via PPI interface.
- **Serial port:** The board is equipped with the RS232 and RS485 serial ports, which are used for parameters transmission between smart sensors and the controller.
- **Ethernet port:** The Ethernet port is included for debugging purposes only. During system debugging, fabric images are compressed and transferred to the PC via an Ethernet cable, then displayed by the PC client software in real-time.
- **Memory:** There are 32 MB data memory and 4 MB program memory on the board.

**Figure 4.**
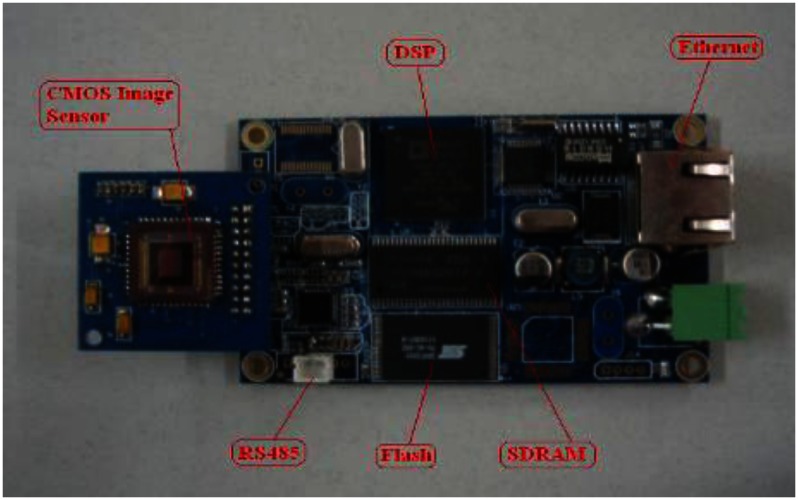
Hardware circuit board of smart visual sensor.

The printed-circuit board of the smart visual sensor is shown in Figure 4.

### 2.3. Software Architecture

**Figure 5.**
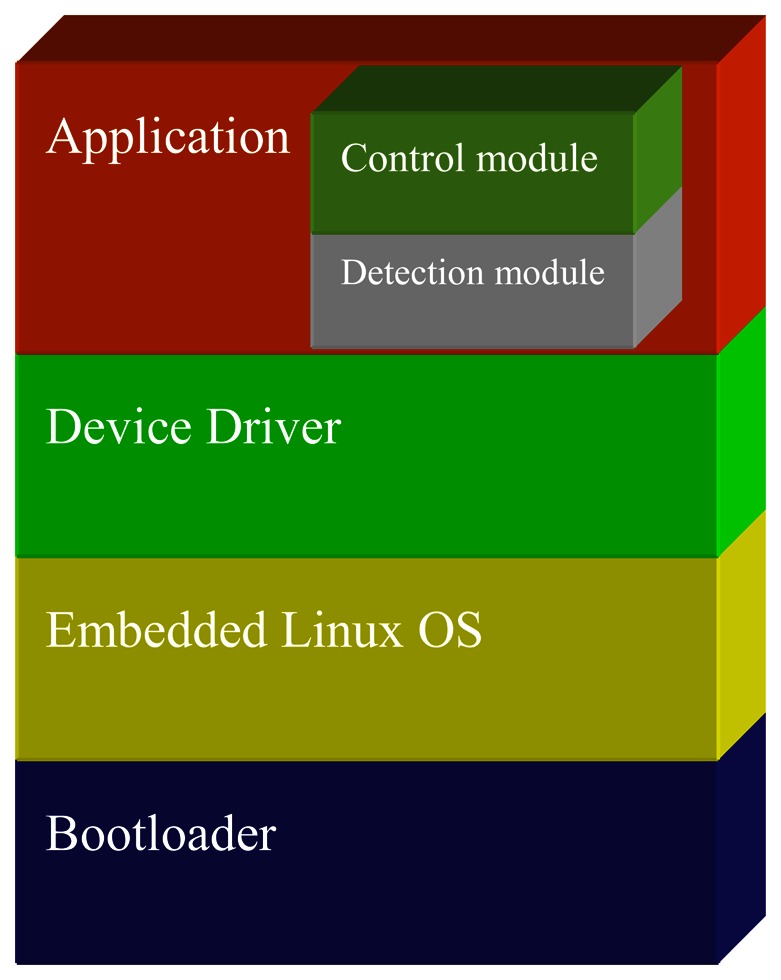
Software architecture of smart visual sensor.

BF537 runs on uClinux OS, and the whole software architecture includes a bootloader, OS, drivers, and application, as shown in Figure 5. The application software is the core part of this architecture, and its work flow mainly contains the following: firstly, original image data are captured from the PPI driver. Secondly, the image data are transmitted to the detection algorithm module for analysis. Finally, the control module commands relay how to operate according to the analysis result.

To meet the real-time demands, we spent a great effort on code optimization until we achieved 10 fps speed. This speed can meet the detection requirements for a warp knitting machine. Most of the optimization works are dependent on processor hardware features, which include the following aspects:

(1) Make good use of internal memory. Level 1 (L1) on-chip memories operate at core clock frequency, and provide high bandwidth and low latency. On-chip memories include SRAM and cache. The code executive speed could be improved greatly by manually putting crucial data and codes into the SRAM. The cache is a kind of internal memory, which manages a processor automatically, and thus, only the crucial codes and data are placed manually into SRAM, while other codes and data just use the cache.
(2) Make full use of DMA operation. Since the DMA controller works in parallel with the CPU, system performance can be greatly improved if we transfer video data from peripherals to memories by DMA while the CPU is simultaneously processing other parts of the data. In fact, the DMA and SRAM are used together. Crucial data are moved from off-chip memories to on-chip memories via DMA first, then computing could start on this block, and at the same time, the DMA begins to move the next block of data.
(3) Execute the most important code via DSP-specific instructions. Specific instructions process video data in multi-media application oriented DSP, such as SAD, pixel add and subtract operations.
(4) Arrange the program code to execute the sequence properly and to improve the cache hit rate via reducing branches and jumps.
(5) Use hardware loop in Blackfin instead of software loop because this hardware loop has a jump mechanism without any CPU payload.
(6) Reduce data access to external memories as much as possible. Since external memories operate using the system clock, a bottleneck will form in the system performance if data access to external memories occurs too frequently.
(7) The memory space of code and data must be allocated carefully. For SDRAM, each bank could open only one page per time, which means each SDRAM could open four pages simultaneously. If the adjacent code and data are stored on the same page, then the extra expense spent on opening and closing pages could be saved. For L1 memory, because there are multiple sub-banks internally, the concurrent access by CPU and DMA controllers is a feasible way to enhance throughout the bandwidth.
(8) Optimize code with assemble language. Blackfin reads 64-bit instructionc each time, so that it is possible to execute several instructions during a single clock cycle with certain constraints.

## 3. Fabric Defect Detection Algorithm

The detection algorithm is the key component in a fabric inspection system. It could run in a PC or in smart visual sensors. Along with the development of computer vision technology during the past two decades, fabric defect detection algorithms have been a popular research topic that has reached several achievements. Ngan [7] summarized the existing results into seven categories, namely, statistics, spectrum analysis, model, learning, structure, hybrid, and motif-based approaches. The spectrum analysis, structure, and other approaches combined with neural networks are the most popular methods nowadays. The spectrum analysis approach includes Fourier, wavelet, Gabor transform, and so on. In Reference [8], Chan applied the Fourier transform to fabric images to detect defects, but locating the defect accurately was hard due to the global feature of the approach. The wavelet transform gained great attention in the fabric detection field because of its good local time-frequency characteristics [9]. This approach performs well on defects with outstanding edges, but poorly on flat defects with smooth grayscale differences. With its good time-frequency properties, the Gabor filter is suitable for emulating the biological features of human eyes and may be used in fabric detection [4,10]. However, since the Gabor filter performs filtering and fusion on multi-scale and multi-direction, which results in heavy computational complexity, meeting real-time requirements is hard [11]. Moreover, this approach is not suitable for flat defects, just like the wavelet approach. The defect area breaks the strong texture background on the fabric image, which is the basic idea for the texture analysis approach. Ojala [12] proposed a texture descriptor based on local binary patterns (LBP), which was applied in fabric defect detection [13,14]. However, LBP just uses space structure information, and ignores grayscale information. In recent years, neural networks were also used for fabric defect detection and classification [15–18], and the typical neural network used for defect detection and classification is the back-propagation (BP) network.

**Figure 6.**
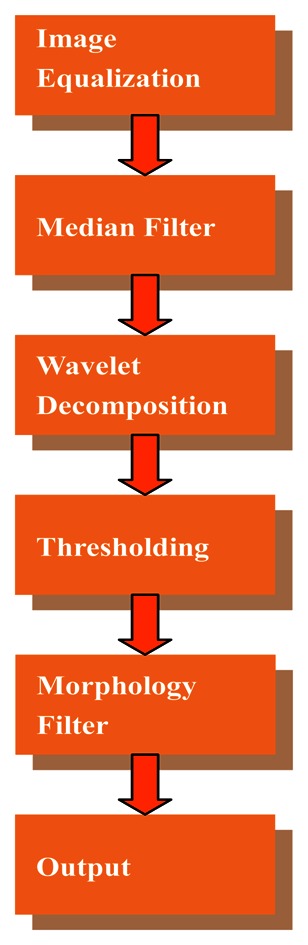
Flow chart of detection algorithm.

Two factors must be considered in choosing the detection algorithm, namely, efficiency and real-time. Among these algorithms, wavelet analysis is very sensitive to line defects and its computational complexity is low, and thus, we chose wavelet analysis as the basis of the detection algorithm. The typical defect on a warp knitting machine is the broken end, *i.e.*, a line defect is caused by a broken warp. On a multi-bar warp knitting machine, the broken end defect caused by a single yarn is not easily seen, especially with the very thin yarn such as 20 D (indicates the weight of 9,000 m of long yarn is 20 grams). Therefore, this kind of defect detection is challenging. To improve detection rate, we propose an improved direct thresholding method on wavelet high frequency coefficients instead of a reconstructed image. In addition, a proper template of mathematic morphology filter is chosen to remove noise, and retain the defect area. The flow chart of our detection algorithm is shown in Figure 6.

### 3.1. Image Preprocess

**Figure 7.**
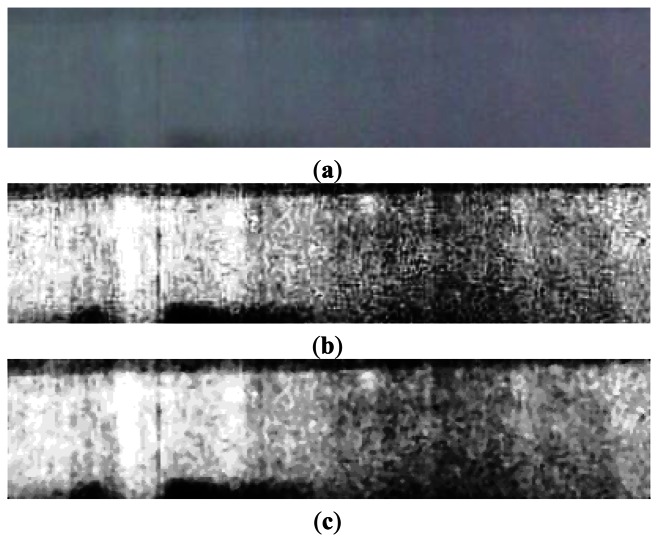
(**a**) Raw image captured by visual sensor; (**b**) Effect of image equalization; and (**c**) Effect of median filtering.

The image captured by the CMOS sensor may be degraded by some factors such as noise or uneven illumination distribution. Therefore, image preprocessing is necessary before analysis to improve image quality. Raw image is equalized firstly, and then noises are removed by median filter with a 3 × 3 template. The raw image, equalization result, and filtering result are shown in Figure 7.

### 3.2. Wavelet Decomposition

Wavelet transform is applied widely in signal analysis due to its excellent local time-frequency characteristics. It offers localized information from horizontal, vertical, and diagonal directions of any input image. The wavelet transform of function *f*(*t*) ∈ *L*^2^(*R*) is defined as:

(1) $$\begin{array}{cc} W_{\Psi ,f}(a,b)={\left|a\right|}^{-1/2}\int_{-\infty }^{+\infty }f(t)•\bar{\psi \left(\frac{t-b}{a}\right)}dt, & a,b\in R,a\neq 0 \end{array}$$

where *ψ*(*t*) ∈ *L*^2^(*R*) and should satisfy $\int_{-\infty }^{+\infty }\frac{{\left|\hat{\psi }(\omega )\right|}^{2}}{\left|\omega \right|}d\omega <\infty$. *ψ̂*(*ω*) is the Fourier transform of *ψ*(*t*). *ψ*(*t*) is called a base wavelet. The inverse wavelet transform is defined by:

(2) $$f(t)=\int_{-\infty }^{+\infty }\int_{-\infty }^{+\infty }\frac{1}{a^{2}}W_{\psi ,f}(a,b)•\psi_{a,b}(t)\text{dadb}$$

where:

(3) $$\psi_{a,b}(t)={\left|a\right|}^{-1/2}\psi \left(\frac{t-b}{a}\right)$$

Performing discretization on Equation (3), we have:

(4) $$\psi_{j,k}(t)={a_{0}}^{-j/2}\psi ({a_{0}}^{-j}t-kb_{0}),\;j,k\in Z$$

Then the corresponding discrete wavelet transform is defined by:

(5) $$W_{\Psi ,f}(j,k)=\int_{-\infty }^{+\infty }f(t)•\bar{\psi_{j,k}(t)}dt$$

**Figure 8.**
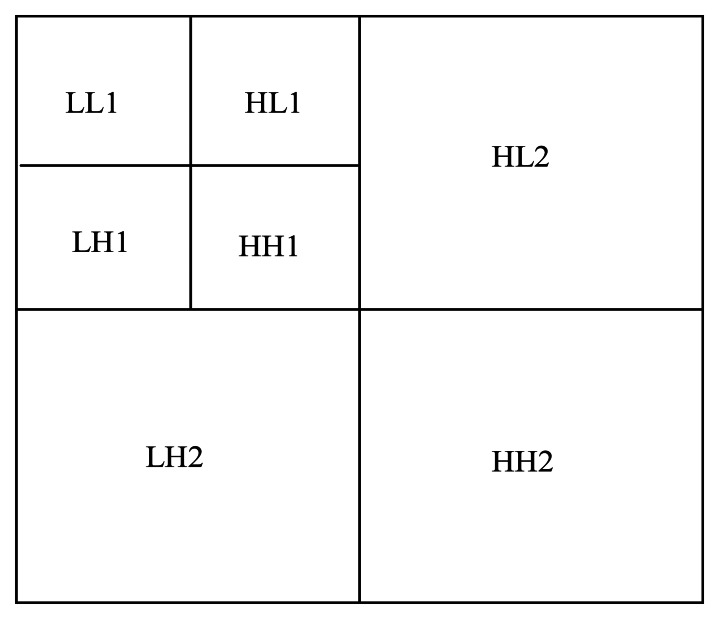
2-Level wavelet decomposition of an image.

The image is decomposed into multiple sub-bands by the wavelet transform. Each sub-band includes different information. The 2-level wavelet decomposition of image is shown in Figure 8. LL is the low frequency coefficient, which denotes the coarse features of the image. LH, HL, and HH are high frequency coefficients of vertical, horizontal, and diagonal directions respectively, which denote the detailed features of the image.

Wong [17] pointed out that the usage of wavelet transforms in fabric defect detection can be divided into two categories, which are textural feature extraction and direct thresholding methods. The wavelet transform is able to extract texture features effectively, and therefore is widely applied to detect defects. The direct thresholding method [9] is based on the concept that the decomposition image of a wavelet can attenuate the texture background and make the defect more obvious. Tsai and Huang [19] proposed a defect detection method based on wavelet reconstruction images. The highlight of their scheme is the inclusion of only the selected sub-bands in the reconstruction process. The selected sub-bands and decomposition level are determined by a factor, which is the energy ratio. Ngan *et al.* [18] presented a direct thresholding method that does not need the reconstruction of the image. The fourth-level horizontal and vertical high frequency coefficients are extracted through the Haar wavelet. Then, the horizontal and vertical sub-bands of level 4 undergo adaptive thresholding. The binarization images of horizontal and vertical are then OR-operated.

The available detection area of web on the warp knitting machine is limited to a narrow rectangle. Thus, only a sub-window of 1,600 × 100 is cropped from the center of the FOV. If we decompose the image four times, the size of sub-bands will very small, and some defect information will be lost. Hence, we only perform 2-level decomposition of the fabric image. The typical defects of the warp knitting machine are very thin linear areas, which have obvious direction features. However, including all the horizontal and vertical high frequency coefficients to locate the defects is not necessary. Based on this premise, we propose an improved direct thresholding method to detect defects. First, the image is decomposed as indicated in Figure 8. Second, the LH2 vertical sub-image undergoes adaptive thresholding. Finally, the binary image is processed by a mathematical morphology filter to obtain the defect areas.

**Figure 9.**
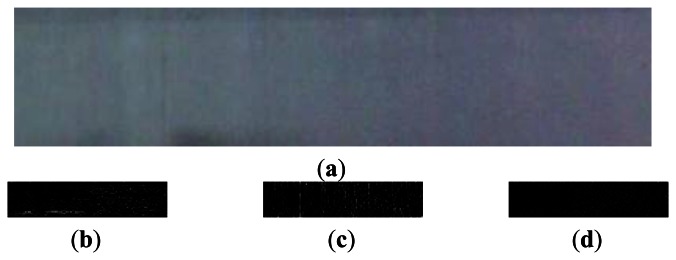
(**a**) Raw image; (**b**) HL2 coefficients; (**c**) LH2 coefficients; and (**d**) HH2 coefficients.

A Daubechies wavelet is employed to perform the 2-level decomposition of the fabric image. After the analysis of several experimental results, we found out that the defects were most outstanding in high frequency coefficients when the vanishing moment was set to 4. The high frequency coefficients after the 2-level decomposition are shown in Figure 9, where HL2 indicates horizontal high frequency coefficients, LH2 refers to vertical high frequency coefficients, and HH2 shows diagonal high frequency coefficients. Because the broken end defects are all vertical on the warp knitting machine, we could obtain outstanding defect features from LH2, while those in HL2 and HH2 are random noises.

### 3.3. Binarization with Adaptive Threshold

**Figure 10.**
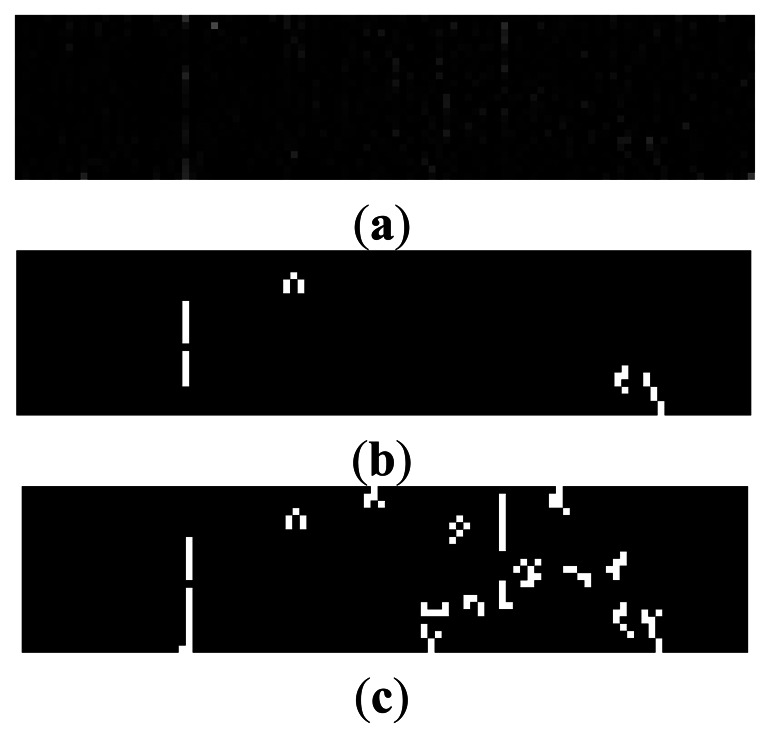
(**a**) LH2 coefficients; (**b**) Binary image; (**c**) Binary image without median filtering.

LH2 sub-image must be segmented by thresholding to get the defect area. The threshold value is selected by using an auto-threshold method. First, an initial value *T* is defined as the average of the highest and lowest grayscale values of pixels. Second, the image is thresholded by *T* into two parts, namely, area *G*_1_ with higher pixel value than *T*, and area *G*_2_ with lower pixel value than *T*. Third, the grayscale average for pixels in *G*_1_ and *G*_2_ are calculated as *u*_1_ and *u*_2_, respectively. Finally, the new threshold value is obtained by the expression (*u*1 + *u*2)/2. This procedure is repeated until the difference of two consequent *T* is within the pre-set range. The binary images after thresholding are shown in Figure 10.

The image captured by CMOS sensor may be degraded by noises which are introduced by circuits or environmental dust. Noise will cause an undesirable result after binarization if median filtering is skipped in the image preprocessing. The binary image without median filtering is shown in Figure 10(c).

### 3.4. Mathematical Morphology Filter

**Figure 11.**
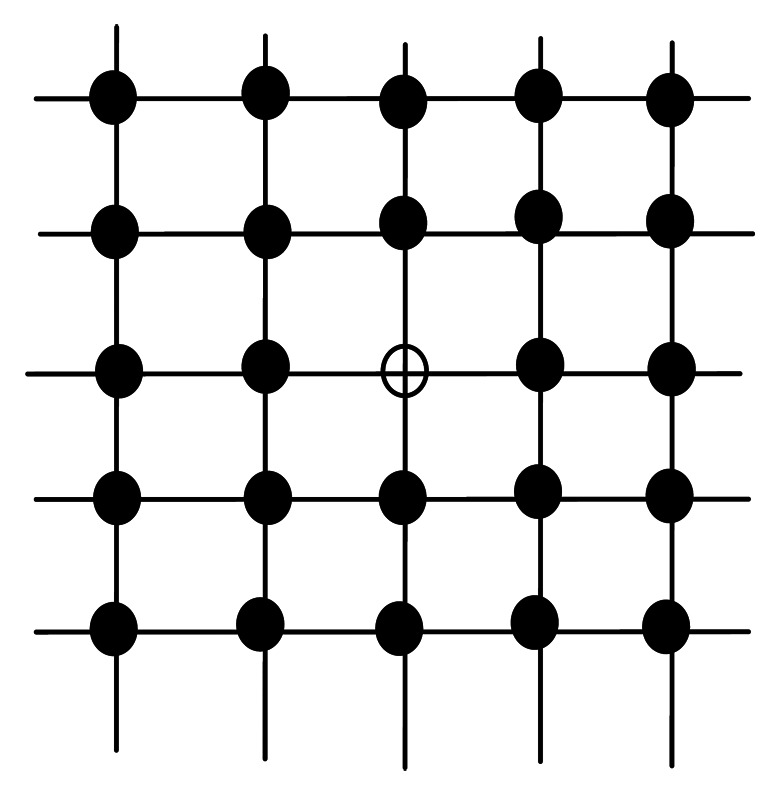
5 × 5 Template.

**Figure 12.**
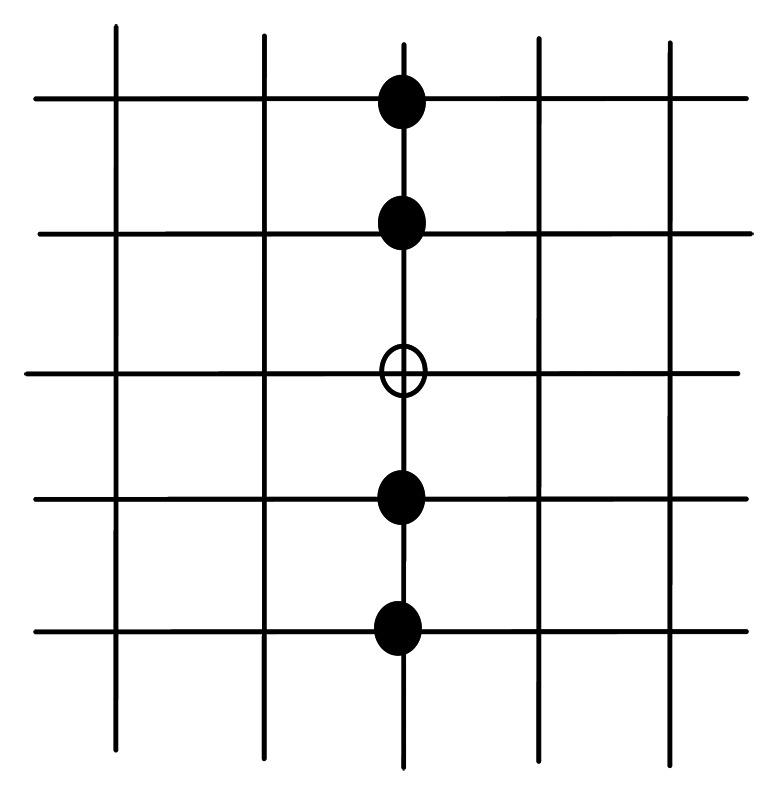
5 × 1 Template.

**Figure 13.**
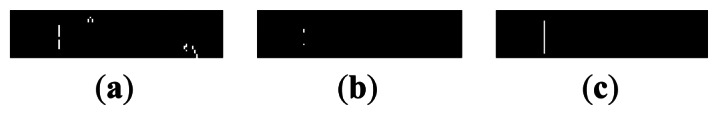
(**a**) Binary image of wavelet coefficients; (**b**) Image after erosion; and (**c**) Image after 2 dilations.

Mathematical morphology is established based on set theory, and can be applied to remove noise from the image. Basic morphology operation includes erosion and dilation. After binarization, defect area and noisy points are white with a pixel value of 255, while other points are black with a pixel value of 0. The traditional morphology template is shown in Figure 11, and 5 × 5 is used for example. The vacant point is the center of template and its value is 255. Only if all the 24 adjacent pixels have the same value as this pixel, then this pixel is set to 255. Otherwise, the pixel value is set to 0. Because defects on warp knitting machine are mostly linear, erosion with traditional *n* × *n* template might erase the defect area as well. Thus, we chose the 5 × 1 vertical template to remove the noise, as shown in Figure 12. Only if the top 2 and bottom 2 pixels are 255, then the vacant pixel is set to 255. According to the experiments, the 5 × 1 template performed well on removing noise. After removing noisy points by erosion, dilation is used to obtain the final defect area. After two dilating operations on eroded images, the discontinuous defect parts are connected, and the results are shown in Figure 13.

## 4. Experiment and Results

**Figure 14.**
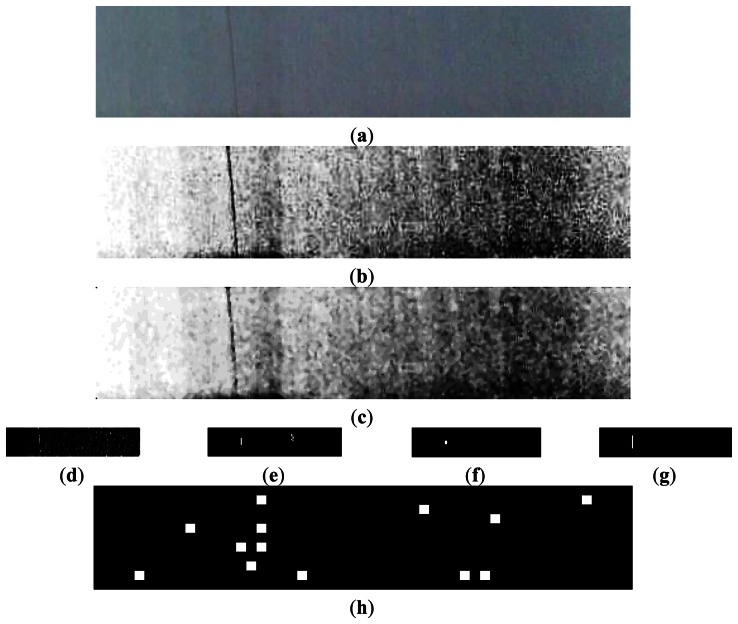
(**a**) Raw image; (**b**) Effect of image equalization; (**c**) Effect of median filtering; (**d**) LH2 wavelet coefficients; (**e**) Binarization image; (**f**) Image after erosion; and (**g**) Image after two dilations; (**h**) Detection result using LBP.

In this section, the experimental results are presented to evaluate the performance of the proposed detection algorithm. The evaluation environment is MATLAB 7.0 and Windows XP was used on a PC. Figure 14(a) shows the test picture with resolution of 424 × 88 cropped from an image captured by the smart visual sensor. There is a very thin linear anomaly embedded in the fabric texture. According to the detection flow of Figure 6, the intermediate and final results are shown in Figure 14(b–g). LBP is another popular method to detect fabric detects. Figure 14(h) is the detection result using LBP [13,14]. It is obvious that LBP couldn't locate the defects accurately. Experimental results show that the proposed method outperforms the LBP algorithm in detecting the defects on the warp knitting machine. The common defects of warp knitting machines include lines and hole defects caused by broken warps. We only give the experimental results of line defects in Figure 14 since detection of hole defects is easier. The most time-consuming step is wavelet transform. But we only perform 2-level decomposition and the wavelet reconstruction is not needed, so the computational complexity is acceptable and reasonable.

## 5. Actual Operation

**Figure 15.**
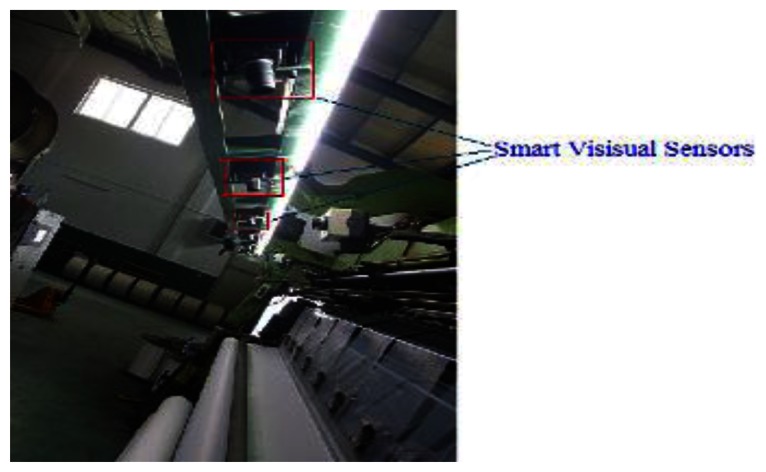
Layout of smart visual sensors for fabric inspection on warp knitting machine.

We developed the application software according to Figures 5 and 6 in C language, and programmed the binary code into flash of the smart visual sensor. Thus, the sensor could run independently on the PC. The proposed fabric defect inspection system based on smart visual sensor was then installed on the warp knitting machines and operated successfully. Smart visual sensors were fixed on the beam over the web, and their position ensured a 90° angle between the lens and fabric surface. There are 6 sensors through the 210-inch-wide web, covering about 800 mm width. The smart visual sensor layout is shown in Figure 15. To eliminate the impacts of light variation, many fluorescent lights are installed parallel with sensors, which are shown in Figure 15. So the illumination is fixed and has no negative effect on quality of image. Additionally, grayscale image is used in our processing algorithm, so color shading or difference also doesn't affect the detection rate.

**Figure 16.**
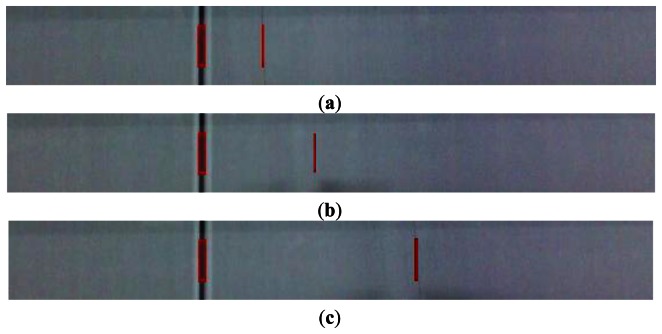
(**a**) Detection result 1; (**b**) Detection result 2; (**c**) Detection result 3.

Each sensor worked independently to deliver the output information of defects, which included coordinates and dimensions. If necessary, the output of sensors and fabric image could be transmitted to PC and displayed by using a PC client software. Figure 16 shows the actual image and detection results, where defects are marked in red rectangles. Program execution cycle is measured in application time running on the DSP. The minimum period is about 100 ms, so the maximal speed is about 10 fps. This processing speed can satisfy the real-time demand of a warp knitting machine.

## 6. Conclusions

Textile quality is traditionally assured by human eyes. However, this manual method has disadvantages of low efficiency and high labor cost. This paper presents a novel automatic inspection system based on smart visual sensors, which was successfully applied to detect defects on a warp knitting machine. Compared with PC-based machine vision systems, our scheme is superior because of its small size, low expense, and high reliability. Since the broken end anomalies on warp knitting machines are very thin, we proposed an improved direct thresholding method based on a wavelet transform to deal with the challenges. Combined with a mathematical morphology filter, a satisfactory detection rate is obtained on the warp knitting machine. The performance of our system has been verified by actual operation in a textile factory. The proposed system has been running for six months and its detection rate can be up to 98%. In our future studies, we will pay attention to detection algorithms for fabric with patterns, and its porting to smart visual sensors.

## References

[1] Abouelela A., Abbas H.M., Eldeeb H., Wahdan A.A., Nassar S.M. (2005). Automated vision system for localizing structural defects in textile fabrics. Pattern Recognit. Lett..

[2] Saeidi M.R.G., Latifi M., Najar S.S., Saeidi A.G. (2005). Computer vision-aided fabric inspection system for on-circular knitting. Text. Res. J..

[3] Furferi R., Governi L. (2006). Development of an artificial vision inspection system for real-time defect detection and classification on circular knitting machines. WSEAS Trans. Comput..

[4] Mak K.L., Peng P. (2008). An automated inspection system for textile fabrics based on gabor filters. Robot. Comput.-Integr. Manuf..

[5] Sun Y., Long H.R. (2011). Adaptive detection of weft-knitted fabric defects based on machine vision system. J. Text. Inst..

[6] Kumar A. (2008). Computer-vision-based fabric defect detection: A survey. IEEE Trans. Ind. Electron..

[7] Ngan H.Y.T., Pang G.K.H., Yung N.H.C. (2011). Automated fabric detect detection-a review. Image Vis. Comput..

[8] Chan C.H., Pang G.K.H. (2000). Fabric defect detection by fourier analysis. IEEE Trans. Ind. Appl..

[9] Ngan H.Y.T., Pang G.K.H., Yung S.P., Ng M.K. (2005). Wavelet based methods on patterned fabric defect detection. Pattern Recognit..

[10] Kumar A., Pang G.K.H. (2002). Defect detection in textured materials using gabor filters. IEEE Trans. Ind. Appl..

[11] Cui L.L., Lu Z.Y., Li J., Li Y.H. (2011). Novel algorithm for automated detection of fabric defect images. J. Xidian Univ..

[12] Ojala T., Pietikäinen M., Mäenpää T. (2002). Multiresolution gray-scale and rotation invariant texture classification with local binary patterns. IEEE Trans. Pattern Anal. Mach. Intell..

[13] Tajeripour F., Kabir E., Sheikhi A. (2007). Defect detection in patterned fabrics using modified local binary patterns. Int. Conf. Comput. Intell. Multimed. Appl..

[14] Liu Z.F., Gao E.J., Li C. (2011). A novel fabric defect detection scheme based on improved local binary pattern operator. Int. Conf. Intell. Syst. Des. Eng. Appl..

[15] Kwak C., Ventura J.A., Karim T.S. (2000). Neural network approach for defect identification and classification on leather fabric. J. Intell. Manuf..

[16] Kumar A. (2003). Neural network based detection of local textile defects. Pattern Recognit..

[17] Wong W.K., Yuen C.W.M., Fan D.D., Chan L.K., Fung E.H.K. (2009). Stitching defect detection and classification using wavelet transform and BP neural network. Expert Syst. Appl..

[18] Qing X.Y., Duan H., Wei J.M., Wang L.J. (2005). A new method to inspect and recognize fabric defects based on wavelet analysis and neural network. Chin. J. Sci. Instrum..

[19] Tsai D.M., Huang T.Y. (2003). Automated surface inspection for statistical textures. Image Vis. Comput..

